# PIKFYVE-dependent regulation of MTORC1 and TFEB

**DOI:** 10.1080/27694127.2022.2082201

**Published:** 2022-06-01

**Authors:** Junya Hasegawa, Ken Inoki, Lois S. Weisman

**Affiliations:** a Life Sciences Institute, University of Michigan, 210 Washtenaw Avenue, Ann Arbor, MI 48109, USA; b Department of Biochemical Pathophysiology, Medical Research Institute, Tokyo Medical and Dental University 1-5-45, Yushima, Bunkyo-ku, Tokyo 113-8510, Japan; c Department of Molecular and Integrative Physiology, University of Michigan Medical School, 1137 East Catherine Street, Ann Arbor, MI 48109, USA; d Department of Internal Medicine, University of Michigan Medical School, 1500 East Medical Center Drive, Ann Arbor, MI 48109, USA; e Department of Cell and Developmental Biology, University of Michigan, 109 Zina Pitcher Place, Ann Arbor, MI 48109

**Keywords:** lysosomes, MTORC1, phosphoinositide lipid, PIKFYVE, protein phosphatase 2A, TFEB

## Abstract

TFEB (transcription factor EB) is essential for the upregulation of gene expression required for macroautophagy/autophagy and lysosomal function. Under nutrient-rich conditions, TFEB is inactivated by MTOR (mechanistic target of rapamycin kinase) complex 1 (MTORC1)-dependent phosphorylation on the lysosome. This suppresses TFEB activity via preventing its translocation to the nucleus. Conversely, under starvation conditions and low MTORC1 activity, MTORC1 sites on TFEB are dephosphorylated, and TFEB translocates to the nucleus, where it activates its transcriptional program. We recently found that the inhibition of PIKFYVE, which produces phosphatidylinositol-3,5-bisphosphate on lysosomes, leads to the dephosphorylation and translocation of TFEB to the nucleus in a manner dependent on PPP2/PP2A (protein phosphatase 2) even under nutrient-rich conditions. Importantly, interaction of TFEB with MTORC1 but not with PPP2 is disrupted when PIKFYVE is inhibited. This suggests that PIKFYVE inhibition results in a loss of contact between MTORC1 and TFEB, which allows PPP2-dependent dephosphorylation of TFEB to be dominant. Interestingly, PIKFYVE-dependent localization of MTORC1 to lysosomes and TFEB phosphorylation requires the RRAG small GTPases. Thus, PIKFYVE may play a critical role in enhancing the activity of RRAG small GTPases, and thereby act as an essential suppressor of TFEB. Therefore, in addition to its direct roles in endosome homeostasis, PIKFYVE may also exert key functions in the regulation of the TFEB-dependent transcriptional program by interacting with the lysosomal nutrient-sensing machinery.

TFEB is an essential transcriptional factor that controls the expression of autophagic and lysosomal genes. The activity of TFEB is largely dependent on its phosphorylation state: MTORC1-dependent phosphorylation of TFEB Ser211 blocks the nuclear translocation of TFEB, thereby inhibiting its cellular transcriptional activity. When MTORC1 is inhibited, Ser211 is dephosphorylated and TFEB translocates to the nucleus where it actives TFEB-dependent gene expression. Phosphorylation at Ser211 can be removed by the protein phosphatase PPP3/calcineurin. Thus, the balance between kinases and phosphatases in the regulation of TFEB is a key element in response to nutrient availability.

MTORC1 is a multiprotein serine/threonine kinase complex and acts as a master regulator of cell growth and proliferation. MTORC1 kinase activity is regulated by various cellular anabolic cues (e.g., amino acids and growth factors) and metabolic stresses. In response to amino acid stimulation, the RRAG small GTPases on the lysosome are activated, which recruits MTORC1 from the cytoplasm to the surface of the lysosome membrane. On the lysosome, MTORC1 is activated by another small GTPase, RHEB, whose activity is mainly stimulated by growth factors. RHEB-induced MTORC1 activation is required for the phosphorylation of many substrates of MTORC1, including RPS6KB1/p70S6K/S6K1, EIF4EBP1, and ULK1 (unc-51 like kinase 1).

Phosphatidylinositol 3,5-bisphosphate (PtdIns[3,5]P_2_), which is among the least abundant phosphoinositides, has critical roles in membrane traffic and ion homeostasis on lysosomes. PIKFYVE, a phosphoinositide 5-kinase, synthesizes PtdIns(3,5)P_2_ from phosphatidylinositol-3-phosphate (PtdIns3P) on endosomes and lysosomes, and is also responsible for the generation of PtdIns5P. Importantly, PIKFYVE controls the volume/size of lysosomes, as well as lysosomal functions.

Our recent study [1] demonstrates that PPP2 stably interacts with TFEB and plays a key role in dephosphorylating Ser211 on TFEB, thus stimulating its nuclear localization and activation. Although PIKFYVE inhibition results in the dephosphorylation and nuclear translocation of TFEB, simultaneous inhibition of PIKFYVE and PPP2 restores phosphorylation of TFEB S221 by MTORC1 and prevents TFEB activation. In contrast, overexpression of PPP2CA (protein phosphatase 2 catalytic subunit alpha), but not its inactive mutant (D85N), induces the dephosphorylation and activation of TFEB even under nutrient-rich conditions. These observations suggest that PPP2 acts downstream of PIKFYVE to regulate MTORC1-dependent TFEB phosphorylation. Note, a previous study also found that PPP2 acts on S211 and activates TFEB during oxidative stress.

Among a dozen direct substrates of MTORC1, TFEB is a unique because RRAG small GTPase activity but not RHEB small GTPase, is critical for MTORC1 phosphorylation of TFEB. Notably, RRAG small GTPases-dependent lysosomal MTORC1 localization and its interaction with TFEB are significantly reduced upon PIKFYVE inhibition. Importantly, reduced TFEB phosphorylation in PIKFYVE-inhibited cells is restored by expressing a constitutively active form of RRAG GTPases, indicating that PIKFYVE acts upstream of RRAG small GTPases for lysosomal MTORC1 localization and phosphorylation of TFEB. Although the molecular mechanisms underlying PIKFYVE-induced RRAG activation and lysosomal MTORC1 localization are unknown, it is tempting to speculate that PIKFYVE-induced accumulation of PtdIns(3,5)P_2_ or PtdIns5P on the lysosome (Figure 1), may play a key role in controlling the activity of regulators of RRAG small GTPases (e.g., GATOR1, KICSTOR, and SLC38A9), which are associated with the lysosomal membrane.

**Figure 1.**
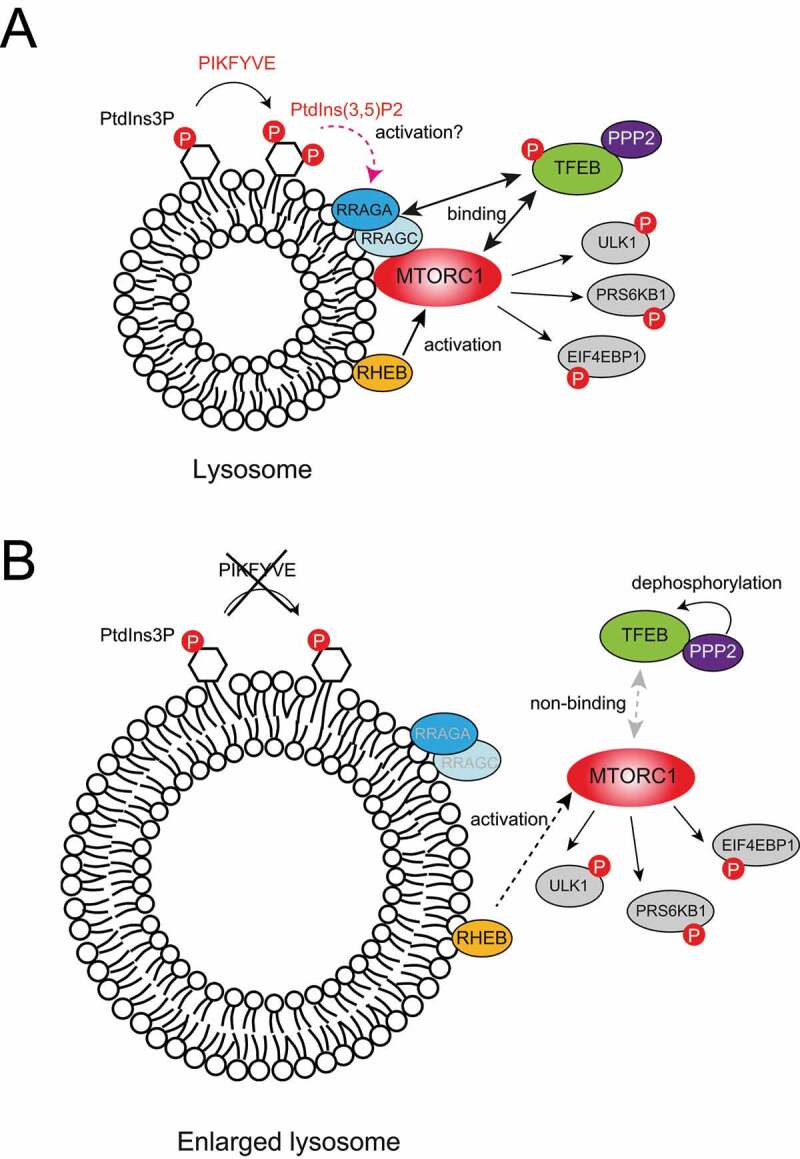
Phosphoinositide turnover controls the MTORC1 signaling on lysosomes. (A) Under nutrient-rich conditions, PIKFYVE induces the interaction between RRAG GTPases and MTORC1 on the surface of lysosomes. Whereas lysosomal MTORC1 phosphorylates TFEB in a manner independent of RHEB, other substrates such as RPS6KB1, EIF4EBP1, and ULK1 require RHEB for MTORC1-dependent phosphorylation. (B) Under PIKFYVE-inhibited conditions, lysosomal MTORC1 recruitment by RRAG small GTPases is decreased, resulting in a significant reduction of MTORC1-dependent TFEB phosphorylation. Despite the reduction of lysosomal MTORC1, PIKFYVE inhibition does not significantly affect RHEB-MTORC1-induced RPS6KB1, EIF4EBP1, or ULK1 phosphorylation, likely due to the enhancement of RHEB activity. PIKFYVE inhibition reduces the interaction between MTORC1 and TFEB, leading to PPP2 phosphatase activity to become the dominant mechanism of TFEB regulation.

Intriguingly, whereas the suppression of PIKFYVE reduces levels of lysosomal MTORC1 this has little effect on the phosphorylation of other canonical MTORC1 substrates, including RPS6KB1 and EIF4EBP1. This may be explained in part by studies reporting that PtdIns(3,5)P_2_ is required for the lysosomal localization of the TSC complex, which acts as a GTPase activating protein/GAP for RHEB, and inhibits RHEB-induced MTORC1 activation. These observations suggest that PIKFYVE may have bidirectional roles in regulating cellular MTORC1 activity.

Other studies indicate that phosphorylation of TFEB by MTORC1 requires the activation of RRAG GTPases and is independent of RHEB GTPase. Ablation of RHEB GTPase abolishes the phosphorylation of RPS6BK1 and EIF4EBP1 but maintains the phosphorylation and cytoplasmic localization of TFEB. These observations may explain why PIKFYVE inhibition inhibits MTORC1-dependent TFEB phosphorylation but does not affect other MTORC1 substrates. In this model, lysosomal RHEB activity is significantly increased upon PIKFYVE inhibition, which may result in a small pool of lysosomal MTORC1 sufficient to phosphorylate other RHEB-dependent MTORC1 substrates such as RPS6BK1 and EIF4EBP1.

The physiological upstream signals for PIKFYVE activation are not fully understood. Glucose starvation or osmotic stress, which inhibit MTORC1 activity, stimulate PIKFYVE activity. Furthermore, PIKFYVE activity is required for autophagosome formation during starvation. Conversely, our new studies suggest that PIKFYVE inhibits gene expression important for autophagy and lysosome biogenesis by enhancing MTORC1-dependent TFEB phosphorylation under nutrient-rich conditions. It remains unclear how PIKFYVE activity is differentially regulated under nutrient-rich or -limited conditions. Elucidating the molecular mechanisms underlying these bidirectional roles of PIKFYVE in the regulation of cellular catabolism and anabolism warrant future investigation.
